# Association of lower urinary tract symptoms and OAB severity with quality of life and mental health in China, Taiwan and South Korea: results from a cross-sectional, population-based study

**DOI:** 10.1186/s12894-017-0294-3

**Published:** 2017-11-21

**Authors:** Kyu-Sung Lee, Tag Keun Yoo, Limin Liao, Jianye Wang, Yao-Chi Chuang, Shih-Ping Liu, Romeo Chu, Budiwan Sumarsono

**Affiliations:** 1 Department of Urology, Samsung Medical Center, Sungkyunkwan University School of Medicine, Seoul, Korea; 2 Department of Urology, Nowon Eulji Medical Center, Eulji University School of Medicine, 68, Hangeulbiseok-ro, Nowon-gu, Seoul, Korea; 3 Department of Urology, China Rehabilitation Research Center, Capital Medical University, Beijing, China; 4 Department of Urology, Beijing Hospital, Beijing, China; 5 Department of Urology, Kaohsiung Chang Gung Memorial Hospital, Chang Gung University College of Medicine, Kaohsiung, Taiwan; 6 Department of Urology, National Taiwan University Hospital and College of Medicine, Taipei, Taiwan; 7 Astellas Pharma Singapore Pte. Ltd., Singapore, Singapore; 8 Present address: 5 Pemimpin Drive, #19-03 Seasons View, Singapore, Singapore

**Keywords:** Asia, Epidemiology, Lower urinary tract symptoms, Mental health, Prevalence, Quality of life

## Abstract

**Background:**

Lower urinary tract symptoms (LUTS) and overactive bladder (OAB) symptoms have a substantial effect on quality of life (QoL). We report QoL and mental health results from a LUTS prevalence study in three Asian countries.

**Methods:**

A cross-sectional, population-representative, internet-based study among individuals aged ≥40 years in China, Taiwan and South Korea. Instruments included: Overactive Bladder Symptom Score (OABSS); International Prostate Symptom Score (IPSS); other International Continence Society (ICS) symptom questions; health-related QoL 12-item short-form (HRQoL-SF12v2); Work Limitations Questionnaire (WLQ); Hospital Anxiety and Depression Scale (HADS). Presence of LUTS was determined according to ICS criteria, with three symptom groups (storage, voiding and post-micturition). Post-stratification weighting matched the age and sex population distribution per country. Initial data analyses were based on descriptive statistics. Significance testing undertaken post hoc included: independent-samples t-test (differences in HRQoL between sexes and between individuals with/without LUTS; relationship between HRQoL score and OABSS; differences in HADS anxiety and depression scores between individuals with/without LUTS; association between HADS anxiety/depression scores and OABSS), chi-square test (association between LUTS prevalence and workplace productivity) and analysis of variance (differences in HRQoL score and in HADS anxiety/depression scores between individuals with different symptom groups, association between HADS anxiety/depression scores and IPSS).

**Results:**

In total, 8284 participants were included. HRQoL scores were significantly worse (*p* < 0.001) among individuals with versus without LUTS (ICS criteria): mean physical health domain scores were 61.1 (standard deviation [SD], 20.1) and 76.7 (17.0), respectively; corresponding mental health domain scores were 34.8 (12.7) and 43.7 (10.7). Workplace productivity was best among individuals without LUTS (difficulties reported by 2–3% of individuals), and worst in those with all three ICS symptom groups (difficulties reported by 29–38% of individuals; *p* = 0.001). Mean HADS scores showed significantly worse (*p* < 0.001) levels of anxiety and depression among individuals with versus without LUTS: anxiety, 6.5 (SD, 3.7) and 4.0 (3.3); corresponding mean depression scores were 6.8 (4.3) and 4.2 (3.6). Increasing OAB severity was also associated with decreasing HRQoL physical and mental health scores.

**Conclusion:**

LUTS and increasing OAB severity are both associated with impaired QoL, reduced workplace productivity, and increased tendency towards anxiety and depression. These results highlight the need to ensure that individuals with LUTS receive appropriate, effective treatment.

**Trial registration:**

ClinicalTrials.gov identifier: NCT02618421, registered 26 November 2015 (retrospectively registered).

## Background

Depending on definition, lower urinary tract symptoms (LUTS) are reported to affect over half of the world’s adult population [1–4]. Although these symptoms are not life-threatening, associations with conditions such as obesity and type 2 diabetes have been reported [5] and they are often bothersome. The potential effects of LUTS are wide-ranging, from impairment of sleep and personal relationships to reductions in emotional well-being and workplace productivity [6]. Consequently, LUTS is associated with impaired quality of life (QoL) [7–9].

Studies from around the world have shown that the impact of LUTS on QoL may be manifested in overall QoL scores, either generic or disease-specific, as well as specific dimensions such as vitality, social functioning, physical activities and mental health [7–16]. The impact of moderate LUTS on QoL has been likened to that of diabetes, hypertension or cancer [12]. LUTS have also been shown in a variety of countries to be associated with increased rates of mental health issues, specifically depression and anxiety [5, 7, 9, 17]. The relationship between LUTS and depressive symptoms appears to be robust regardless of sex/ethnicity [18].

The substantial impact of LUTS on QoL reinforces the need for their treatment, and indicates the potential benefits of effective intervention. In addition, treatment of LUTS has been shown to improve QoL [19]. However, many patients with LUTS do not seek healthcare [20] and LUTS therapy may not be regarded as a high priority by primary care physicians [21].

We conducted a study to determine the prevalence of LUTS in the population aged ≥40 years in China, Taiwan and South Korea, using symptom definitions approved by the International Continence Society (ICS) in 2002 [22]. Here we report QoL and mental health results from the study.

## Methods

As the study methods are published in full elsewhere [23], they are described here in brief.

### Study design and population

We conducted a cross-sectional, population-representative internet-based study in China, Taiwan and South Korea. Inclusion criteria were age ≥ 40 years, internet access and ability to read the local language. Pregnant women and individuals with a urinary tract infection during the previous month were excluded. The study was performed in compliance with the principles of the Declaration of Helsinki, Good Clinical Practice and the World Association for Social, Opinion and Market Research (ESOMAR) guidelines [24]. Informed consent was obtained from all participants.

Consumer survey panels were actively managed to ensure random sampling with representation of the target population in terms of age, sex and socioeconomic factors.

### Endpoints

Instruments in the study were validated in the local language and included the following: the International Prostate Symptom Score (IPSS) [25]; other ICS symptoms questions (related to splitting/spraying, hesitancy, terminal dribble, urgency); Overactive Bladder Symptom Score (OABSS) [26]; the 12-item short-form health survey for measuring health-related QoL (HRQoL-SF12v2; possible scores for mental health domain and physical health domain range from 0 to 100, with higher scores indicating better health) [27]; Work Limitations Questionnaire (WLQ) [28]; Hospital Anxiety and Depression Scale (HADS; total score for both anxiety and depression classified as normal, 0-7, borderline abnormal, 8-10, or abnormal, 11-21) [29]. Presence of LUTS was based on ICS criteria (presence of voiding, storage or post-micturition symptom[s] with frequency ≥ 1 in 5 times), with the exception that nocturia was defined as ≥2 episodes per night (ICS definition for nocturia is ≥1 episode per night; the higher threshold was chosen to avoid over-estimation) [22].

### Statistical analysis

A minimum sample size of 384 respondents per group was needed for estimating LUTS affecting 50% of patients within five percentage points. Five different age groups (40–44, 45–49, 50–54, 55–60 and >60 years) were planned for analysis, necessitating 1920 individuals per country. With an assumption that ~28% of data would be non-evaluable, a total population of 8000 study participants was planned (4000 in China, 2000 in Taiwan and 2000 in South Korea). The initial data analyses were based on descriptive statistics. Workplace productivity analyses excluded individuals who selected ‘does not apply to my job’ as a response. Post-stratification weighting was performed to match the age and sex distributions of the populations in the respective countries. All significance testing was undertaken post hoc. Predictors of HADS scores were identified by logistic regression. The independent-samples t-test was used for the following: differences in HRQoL scores between men and women; differences in HRQoL scores between individuals with or without LUTS according to ICS criteria; relationship between HRQoL score and severity of overactive bladder (OAB) according to the OABSS; differences in HADS anxiety and depression scores between individuals with or without LUTS according to ICS criteria; and the relationship between HADS anxiety and depression scores and OABSS. Associations between LUTS prevalence according to ICS criteria and workplace productivity were examined using the chi-square test. Analysis of variance (ANOVA) was used to assess differences in HRQoL score between individuals with different ICS symptom groups, and differences in HADS anxiety and depression scores between individuals with different ICS symptom groups. The relationships between HADS anxiety and depression scores and severity of OAB according to IPSS were also assessed by ANOVA.

## Results

The survey sample and response rate for each country has been reported previously [23]. The study included a total of 8284 participants, of whom 4136 were from China, 2068 from Taiwan and 2080 from South Korea. Table 1 shows demographic characteristics of the population.

**Table 1:** Participants’ demographic data. Table reproduced from Chapple et al. 2017 [23]

	China (*n* = 4136)	Taiwan (*n* = 2068)	South Korea (*n* = 2080)	Overall (*n* = 8284)
Sex				
Men	50.3%	48.6%	47.6%	49.2%
Women	49.7%	51.4%	52.4%	50.8%
Age group				
40–44 years	19.9%	15.6%	16.9%	18.1%
45–49 years	19.6%	16.1%	16.8%	18.0%
50–54 years	15.3%	16.3%	16.3%	15.8%
55–59 years	12.7%	14.9%	14.4%	13.7%
≥ 60 years	32.6%	37.1%	35.6%	34.4%
Education				
High school or less	28.0%	39.3%	30.3%	31.4%
Some college	28.4%	23.7%	3.4%	20.9%
College degree/college graduate	40.2%	28.2%	57.0%	41.4%
Postgraduate	3.5%	8.8%	9.4%	6.3%
Marital status				
Single	2.9%	14.9%	9.2%	7.5%
Divorced	1.5%	5.2%	4.0%	3.0%
Married/living with partner	91.7%	72.8%	81.6%	84.5%
Widow/widower	3.2%	6.3%	4.5%	4.3%
Prefer not to answer	0.8%	0.7%	0.7%	0.7%
Work status				
Homemaker	2.6%	12.0%	22.4%	9.9%
Retired	28.7%	16.9%	6.0%	20.1%
Student	0.0%	0.1%	0.2%	0.1%
Working, full-time	62.6%	60.0%	53.9%	59.8%
Working, part-time	3.7%	6.2%	8.0%	5.4%
Other work for pay	0.4%	0.8%	3.2%	1.2%
Other	1.1%	1.9%	3.7%	2.0%
Unemployed	0.6%	1.3%	2.3%	1.2%
Permanently disabled/cannot work due to ill health	0.1%	0.8%	0.4%	0.4%

**Fig. 1:**
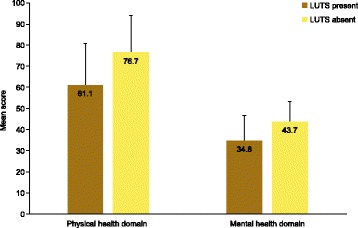
HRQoL scores (assessed using the 12-item short-form health survey) among individuals with or without LUTS according to ICS criteria. *HRQoL* health-related quality of life, *ICS* International Continence Society, *LUTS* lower urinary tract symptoms

HRQoL scores were lower (i.e. worse) among individuals with versus without LUTS according to ICS criteria (Fig. 1; *p* < 0.001 for both physical health domain and mental health domain). For the overall population with and without LUTS, the mean physical health domain scores were 61.1 (standard deviation [SD], 20.1) and 76.7 (17.0), respectively, and the mean mental health domain scores were 34.8 (12.7) and 43.7 (10.7). These differences were evident in all three countries. Men had higher (i.e. better) HRQoL scores than women, for both physical and mental health domains (*p* < 0.001 for both), but LUTS were associated with similar score reductions in both sexes. Individuals with all three ICS symptom groups had the lowest mean HRQoL scores (physical health domain: 52.3 [SD, 18.5]; mental health domain: 29.4 [11.5]; *p* < 0.001 for both domains versus all other ICS symptom group combinations), and the presence of two symptom groups was generally associated with lower HRQoL scores than one (Table 2). The presence of voiding and storage symptoms was associated with greater HRQoL impairment than any other pair of ICS symptom groups, with a mean physical health domain score of 58.4 (20.0) and a mean mental health domain score of 33.8 (13.2). Increasing severity of OAB, according to OABSS, was associated with decreasing HRQoL physical and mental health scores (Table 2; *p* < 0.001 for individuals with versus without OAB). These trends were similar in China, South Korea and Taiwan.

**Table 2:** HRQoL by LUTS and OABSS

	Physical health domain		Mental health domain	
	Mean score (standard deviation)^a^	*p*-value for comparison vs. No LUTS or No OAB	Mean score (standard deviation)^a^	*p*-value for comparison vs. No LUTS or No OAB
LUTS (ICS criteria)				
No LUTS	76.7 (17.0)	N/A	43.7 (10.7)	N/A
Voiding Only	71.5 (17.4)	<0.005	40.9 (10.9)	<0.005
Storage Only	68.2 (18.5)	<0.005	38.9 (12.0)	<0.005
PM Only	68.2 (18.0)	<0.005	38.6 (12.5)	<0.005
Voiding + Storage	58.4 (20.0)	<0.005	33.8 (13.2)	<0.005
Voiding + PM	68.8 (17.9)	<0.005	39 (12.0)	<0.005
Storage + PM	63.6 (19.0)	<0.005	36.2 (11.6)	<0.005
Voiding + Storage + PM	52.3 (18.5)	<0.005	29.4 (11.5)	<0.005
OABSS				
No OAB	71.4 (18.7)	N/A	40.8 (11.8)	N/A
Mild OAB	58.6 (18.2)	<0.0005	33.3 (11.6)	<0.0005
Moderate OAB	47.7 (16.8)	<0.0005	26.7 (10.7)	<0.0005
Severe OAB	33.6 (15.6)	<0.0005	18.9 (9.5)	<0.0005
Overall	67.1 (20.4)		38.2 (12.8)	

*ICS* International Continence Society, *LUTS* lower urinary tract symptoms, *OAB* overactive bladder, *OABSS* Overactive Bladder Symptom Score, *PM* post-micturition

^a^ data are for both sexes and all three countries combined

The presence of LUTS according to ICS criteria was associated with statistically significantly impaired workplace productivity (Table 3; *p* = 0.001 for all eight domains, LUTS present versus LUTS absent). The best productivity was evident among individuals without LUTS, while productivity was worst in those with all three ICS symptom groups. Second worst results were obtained from individuals with voiding and storage symptoms. Compared with individuals without LUTS, productivity was least affected among individuals with only one ICS symptom group. The results showed a similar pattern across all eight items of the work limitations questionnaire. Impairment of workplace productivity (all eight items) was shown to increase with increasing severity of OAB (Table 3; *p* = 0.001 for all eight domains, OAB present versus OAB absent).

**Table 3:** Workplace productivity by LUTS and OABSS

	Number of participants (%) for the overall population						
	Difficult to get going easily at start of work day^a, b^	Difficult to start on your job as soon as you arrived at work^a, b^	Able to sit, stand, or stay in one position for longer than 15 min while working, without difficulty^a, c^	Able to repeat the same motions over and over again while working, without difficulty^a, c^	Difficult to speak with people in-person, in meetings or on the phone ^a, b^	Difficult to handle the workload ^a, b^	Difficult to finish work on time ^a, b^
LUTS (ICS criteria)							
No LUTS	60 (3%)	57 (2%)	954 (40%)	967 (40%)	45 (2%)	53 (2%)	66 (3%)
PM Only	13 (8%)^†^	8 (5%)	58 (37%)	64 (42%)	8 (5%)*	9 (6%)*	7 (4%)
Storage + PM	26 (11%)^†^	23 (10%)^†^	89 (38%)	81 (35%)	16 (7%)^†^	17 (7%)^†^	24 (10%)^†^
Storage Only	98 (6%)^†^	98 (6%)^†^	566 (38%)	564 (37%)	98 (7%)^†^	94 (6%)^†^	106 (7%)^†^
Voiding + PM	11 (6%)*	9 (5%)	75 (40%)	74 (39%)	8 (4%)	10 (5%)*	13 (7%)*
Voiding + Storage	183 (22%)^†^	178 (21%)^†^	307 (37%)	293 (35%)*	166 (20%)^†^	152 (18%)^†^	169 (20%)^†^
Voiding + Storage + PM	640 (36%)^†^	645 (36%)^†^	590 (33%)^†^	543 (31%)^†^	557 (31%)^†^	511 (29%)^†^	559 (31%)^†^
Voiding Only	9 (2%)	10 (3%)	130 (35%)	125 (34%)*	11 (3%)	14 (4%)	17 (4%)
OABSS							
No-OAB	401 (7%)	365 (6%)	2150 (38%)	2142 (37%)	334 (6%)	331 (6%)	374 (7%)
Mild-OAB	114 (20%)^†^	112 (19%)^†^	223 (39%)	212 (37%)	88 (15%)^†^	89 (16%)^†^	97 (17%)^†^
Moderate-OAB	485 (45%)^†^	506 (47%)^†^	376 (35%)	344 (32%)^†^	446 (41%)^†^	400 (37%)^†^	446 (41%)^†^
Severe-OAB	40 (62%)^†^	45 (69%)^†^	19 (29%)	16 (25%)*	43 (66%)^†^	39 (60%)^†^	42 (65%)^†^
Overall	1040 (14%)	1027 (14%)	2768 (37%)	2713 (36%)	910 (12%)	859 (12%)	960 (13%)

Numbers of individuals are weighted and rounded, therefore category totals may not equal population totals shown in column headings. Percentages are based on the weighted ‘*n*’ values

*ICS* International Continence Society, *LUTS* lower urinary tract symptoms, *OAB* overactive bladder, *OABSS* Overactive Bladder Symptom Score, *PM* post-micturition

^a^ Because of physical health or emotional problems; excludes those who responded that this did not apply to their job

^b^ ≥ ~50% of the time

^c^ < ~50% of the time

*p < 0.05 vs. No LUTS or No OAB

^†^ *p* < 0.005 vs. No LUTS or No OAB

**Fig. 2:**
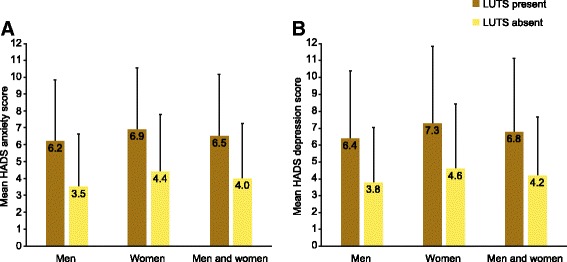
HADS by LUTS according to ICS criteria: **a** anxiety and **b** depression. *HADS* Hospital Anxiety and Depression Scale, *ICS* International Continence Society, *LUTS* lower urinary tract symptoms

Individuals with LUTS according to ICS criteria had higher (i.e. worse) scores for both anxiety and depression compared with those without LUTS (Fig. 2; *p* < 0.001 for men, women and both sexes together). The mean anxiety score for the population with LUTS was 6.5 (SD, 3.7), compared with 4.0 (3.3) for the population without LUTS. The corresponding mean depression scores were 6.8 (4.3) and 4.2 (3.6). Women had higher HADS scores than men (*p* < 0.0001 for both anxiety and depression), but the difference between individuals with versus without LUTS was similar in both sexes (Table 4). The highest (i.e. worst) HADS scores were observed among those with all three ICS symptom groups (*p* < 0.001 versus all other ICS symptom group combinations), with mean scores for anxiety and depression of 8.0 (3.6) and 8.2 (4.3), respectively. Individuals with two symptom groups generally had higher HADS scores than those with one symptom group. HADS scores were also higher among individuals with versus without OAB (*p* < 0.0001 for both anxiety and depression). HADS scores increased markedly with increasing OAB severity assessed by OABSS: mean anxiety scores for individuals with no versus severe OAB were 4.9 (3.5) and 11.5 (4.3); the corresponding depression scores were 5.1 (4.0) and 11.5 (5.1). In addition, statistically significant relationships were observed between HADS scores and IPSS-measured symptom severity (increased IPSS severity associated with higher HADS anxiety and depression scores; *p* < 0.001). The associations between HADS scores and OABSS/IPSS severity were evident in both men and women. Statistically significant predictors of high HADS scores (≥8, indicating clinically relevant levels of anxiety and depression [30]) are shown in Table 5. Urgency with fear of leaking and stress incontinence (different causes) were associated with high depression and anxiety scores in both men and women. Incomplete emptying was a predictor of high scores for anxiety and depression in women, while perceived frequency and terminal dribble were predictors of high scores for both parameters in men as well as anxiety in women.

**Table 4:** HADS by LUTS, IPSS and OABSS

	Anxiety score^a^			Depression score^a^		
	Men	Women	Men and women	Men	Women	Men and women
LUTS (ICS criteria)						
No LUTS	3.5 (3.1)	4.4 (3.4)	4.0 (3.3)	3.8 (3.3)	4.6 (3.9)	4.2 (3.6)
Voiding Only	4.8 (3.4)^†^	5.3 (3)	4.9 (3.3)^†^	5.5 (4)^†^	6.3 (4.2)^†^	5.6 (4)^†^
Storage Only	5.1 (3.4)^†^	5.7 (3.4)^†^	5.5 (3.4)^†^	5.2 (3.8)^†^	6 (4.1)^†^	5.7 (4)^†^
PM Only	4.6 (3.1)	5.8 (3.1)*	5.1 (3.2)^†^	5 (3.4)	6.7 (4.4)^†^	5.7 (3.9)^†^
Voiding + Storage	6.1 (3.5)^†^	7 (3.6)^†^	6.5 (3.6)^†^	6.4 (3.8)^†^	7.4 (4.3)^†^	6.9 (4.1)^†^
Voiding + PM	5.1 (3.5)^†^	6.6 (3.6)^†^	5.4 (3.5)^†^	5.8 (3.9)^†^	7.7 (4.9)^†^	6.2 (4.2)^†^
Storage + PM	6 (3.4)^†^	6.7 (3.3)^†^	6.4 (3.4)^†^	6.1 (3.8)^†^	7.3 (4.4)^†^	6.7 (4.1)^†^
Voiding + Storage + PM	7.5 (3.6)^†^	8.7 (3.5)^†^	8.0 (3.6)^†^	7.5 (3.9)^†^	9.2 (4.6)^†^	8.2 (4.3)^†^
OABSS						
No OAB	4.5 (3.5)	5.2 (3.5)	4.9 (3.5)	4.8 (3.7)	5.5 (4.2)	5.1 (4.0)
Mild OAB	6.6 (3.5)^†^	7.3 (3.2)^†^	6.9 (3.4)^†^	7.1 (3.7)^†^	7.7 (4)^†^	7.4 (3.9)^†^
Moderate OAB	8.2 (3.4)^†^	8.7 (3.5)^†^	8.5 (3.5)^†^	8.1 (3.8)^†^	9.3 (4.6)^†^	8.7 (4.3)^†^
Severe OAB	10.9 (4.3)^†^	11.9 (4.4)^†^	11.5 (4.3)^†^	10.7 (3.6)^†^	12.1 (5.9)^†^	11.5 (5.1)^†^
IPSS						
No Symptom	3 (3.2)	3.8 (3.5)	3.4 (3.4)	3.5 (3.5)	4.5 (4)	4 (3.8)
Mild	4.4 (3.3)^†^	5.2 (3.4)^†^	4.8 (3.4)^†^	4.6 (3.5)^†^	5.3 (4)^†^	5 (3.8)^†^
Moderate	6.5 (3.4)^†^	7.5 (3.3)^†^	6.9 (3.4)^†^	6.8 (3.8)^†^	8 (4.4)^†^	7.3 (4.1)^†^
Severe	9.2 (3.9)^†^	10 (3.7)^†^	9.6 (3.9)^†^	9.1 (4.1)^†^	10.4 (4.7)^†^	9.8 (4.4)^†^
Overall	5.2 (3.7)	5.9 (3.8)	5.5 (3.8)	5.4 (3.9)	6.2 (4.5)	5.8 (4.2)

*HADS* Hospital Anxiety and Depression Scale, *ICS* International Continence Society, *IPSS* International Prostate Symptom Score, *LUTS* lower urinary tract symptoms, *OAB* overactive bladder, *OABSS* overactive bladder symptom score, *PM* post-micturition

^*^ *p* < 0.05 vs. No LUTS, No OAB or No Symptom

^†^ *p* < 0.005 vs. No LUTS, No OAB or No Symptom

^a^ mean scores (standard deviation) for all three countries combined

**Table 5:** Significant predictors of HADS scores ≥8

	Men	Women
HADS anxiety score	Voiding symptoms: Straining, terminal dribble Storage symptoms: Perceived frequency, nocturia, urgency with fear of leaking, and stress incontinence (in relation to sneezing, exercising, nocturnal enuresis or sexual activity)	Voiding symptoms: Terminal dribble Storage symptoms: Perceived frequency, nocturia, urgency with fear of leaking, and stress incontinence (nocturnal enuresis) Post-micturition symptoms: Incomplete emptying
HADS depression score	Voiding symptoms: Terminal dribble Storage symptoms: Perceived frequency, urgency, urgency with fear of leaking, urgency incontinence (how often), and stress incontinence (in relation to coughing or sexual activity)	Storage symptoms: Nocturia, urgency with fear of leaking, and stress incontinence (in relation to laughing or for no reason) Post-micturition symptoms: Incomplete emptying

*HADS* Hospital Anxiety and Depression Scale

## Discussion

This study provides strong evidence that LUTS are associated with impaired QoL, reduced workplace productivity, and increased tendency towards anxiety and depression. These findings are evident in both men and women from all three countries included in the study. Individuals with all three ICS symptom groups showed greater impairment than those with two ICS symptom groups, and the presence of two ICS symptom groups was associated with greater impairment than one symptom group. Voiding and storage were associated with greater QoL impairment than other pairs of symptom groups. Workplace productivity decreased with increasing severity of OAB, while HADS scores deteriorated with increasing OAB and IPSS severity. Urgency with fear of leaking was a significant predictor of high HADS scores for anxiety and depression in both men and women.

Overall, our results are consistent with data from previous studies assessing the impact of LUTS on QoL and mental health in countries outside Asia. In the EpiLUTS study, conducted in 30,000 adults aged ≥40 years in Sweden, UK and USA, similar methods to the current study were used for assessing LUTS (ICS criteria), HRQoL (SF-12) and mental health (HADS) [9]. As in our study, deteriorations in physical and mental components of the SF-12, as well as anxiety and depression scores, were most pronounced among individuals with all three ICS symptom groups. A study of urinary incontinence in women from France, Germany, UK and USA (*N* = 1203) showed that the impact of symptoms on HRQoL (measured using the International Consultation on Incontinence Modular Questionnaire Lower Urinary Tract Symptoms Quality of Life [ICIQ-LUTSqol]) increased with increasing symptom severity [6]. Evidence that QoL impairment increases with symptom severity was also provided by a Mexican study conducted in a population aged ≥70 years (*N* = 1124): individuals with severe urinary incontinence had worse self-perceived health status and greater disability than those with less severe symptoms [10]. In addition, this study reported increased symptoms of depression among those with severe incontinence. Data from the UREPIK and BACH studies, which were performed in men aged 40–79 years (*N* = 6486) from five cities (Boxmeer, the Netherlands; Auxerre, France; Birmingham, UK; Seoul, South Korea and Boston, USA), also showed that QoL decreased with increasing severity of LUTS [12]. A 10-point increase in IPSS was associated with a 3.3-point reduction in SF-12 physical health component score, and a 1.4–3.4-point reduction in the mental health component score [12]. A US study reported that storage but not voiding symptoms was significantly associated with anxiety and depression [5]. Our study showed some trends towards greater increases in anxiety and depression scores among individuals with storage versus voiding symptoms, but the differences were small.

Studies conducted in Asian populations also reported similar findings to our study. South Korean women with OAB or stress urinary incontinence (SUI) have been shown to have lower quality of life (i.e. higher scores for all King’s Health Questionnaire domains) than controls [15]. The same study reported lower Short Form-36 (SF-36) scores versus controls for four out of eight domains in women with OAB, and for one domain in women with SUI. A door-to-door survey of South Korean men aged ≥40 years showed that generic health status and workplace productivity were impaired among individuals with LUTS compared with those without LUTS [7]. Increased symptoms of major depression were also observed in men with LUTS. A third South Korean study involved 625 men and women with OAB [31]. Increasing severity of incontinence was associated with significantly lower QoL (measured using the Incontinence-Specific Quality of Life Instrument), increased symptom bother, poorer health-related utility (according to EQ-5D), increased expenditure on incontinence pads, and increased interference with work and regular activities. In the same study, frequency, urgency and nocturia were independently associated with QoL impairment [31]. In Taiwanese women with SUI, significant correlations between the severity of incontinence and incontinence-related QoL have been observed [14]. Another study of Taiwanese women (age range: 35–64 years; *N* = 4661) reported reduced SF-36 scores, including physical and mental components, among individuals with urinary incontinence [8]. This study also showed that urinary incontinence had a greater impact on mental health-related HRQoL than diabetes, hyperlipidaemia, and chronic kidney disease. A third Taiwanese study showed that women with mixed urinary incontinence had lower QoL than those with urge incontinence or stress incontinence [32]. In China, HRQoL was assessed in individuals with LUTS and compared with data for the normal population [33]. Reduced scores were observed among the population with LUTS for the general health and vitality domains and for the physical component, although LUTS was associated with a higher role emotion domain score. HRQoL impairment increased with increased LUTS severity. In another Chinese study, data from >1000 adults showed that increasing episodes of nocturia was an independent predictor of impaired nocturia-related QoL [16].

Our findings also reflect previous Asian mental health data. In Taiwan, the prevalence of depression or anxiety has been found to be twice as high among individuals with LUTS versus matched controls (11.45% vs. 5.72%) [34]. Similarly, the odds ratio of depression in Korean men with versus without LUTS has been reported to be 2.87 [35]. A study performed in Hong Kong reported that the relationship between LUTS and depressive symptoms is robust after adjustment for other factors associated with depression such as divorce, cardiac disease and smoking [36].

Our study suggests considerable scope to reduce the overall burden of LUTS by increasing the percentage of patients who consult healthcare professionals, which will help patients gain access to the most effective available treatment for their condition. A variety of treatments that can be prescribed for LUTS have been shown to improve patients’ QoL and/or mental health (e.g. drug treatment such as alpha blockers or antimuscarinics, surgical options such as transobturator tape or transurethral resection, botulinum toxin injections) [37–46].

The survey population is an important strength of our study. Younger individuals were not included because of previous data showing that LUTS are highly prevalent above the age of 40 [2] and numerous other epidemiological studies have focused on populations aged ≥40 years [6, 7, 9, 12, 47]. Additional strengths include the large number of participants and the use of well-established instruments to determine the presence and severity of LUTS and their effects on QoL and mental health. Our survey was conducted in countries with the highest internet penetration rates in Asia (South Korea, 92%; Taiwan, 84% and China 52%) [48]. Use of the internet to conduct a survey encourages full and honest responses to sensitive questions – there can be a tendency for biased answers when questions are asked by an interviewer [49]. On the other hand, we cannot be certain that results among individuals without internet access would be the same as those reported here. Also, when completing a questionnaire online, study participants may potentially interpret questions differently from those asked by a healthcare professional as interviewer. Although our results are similar to other studies around the globe, our study is limited by statistical analyses being undertaken post hoc; ideally these should have been identified a priori. The study was not designed to assess costs associated with LUTS, although impairment of workplace productivity indicates a financial impact and a health economic evaluation may have been useful. Previous investigations have shown that the economic burden of LUTS is significant (e.g. estimated annual costs up to $32 billion in the USA) [50, 51].

## Conclusions

In conclusion, this international study demonstrates the association of both LUTS and increasing OAB severity with impairment of QoL, workplace productivity and mental health in three Asian countries. These results are consistent with previous studies, and highlight the need to ensure that individuals with LUTS consult healthcare professionals to receive appropriate and effective treatment.

## Abbreviations

ANOVA: Analysis of variance
HADS: Hospital anxiety and depression scale
HRQoL-SF12v2: 12-item short-form health survey for measuring health-related quality of life
ICIQ-LUTSqol: International Consultation on Incontinence Modular Questionnaire Lower Urinary Tract Symptoms Quality of Life
ICS: International Continence Society
IPSS: International Prostate Symptom Score
LUTS: Lower urinary tract symptoms
OABSS: Overactive bladder symptom score
QoL: Quality of life
SD: Standard deviation
WLQ: Work limitations
